# Nutritional and Hormonal Regulation of Citrate and Carnitine/Acylcarnitine Transporters: Two Mitochondrial Carriers Involved in Fatty Acid Metabolism

**DOI:** 10.3390/ijms17060817

**Published:** 2016-05-25

**Authors:** Anna M. Giudetti, Eleonora Stanca, Luisa Siculella, Gabriele V. Gnoni, Fabrizio Damiano

**Affiliations:** Laboratory of Biochemistry and Molecular Biology, Department of Biological and Environmental Sciences and Technologies, University of Salento, Lecce 73100, Italy; anna.giudetti@unisalento.it (A.M.G.); eleonora.stanca@unisalento.it (E.S.); gabriele.gnoni@unisalento.it (G.V.G.); fabrizio.damiano@unisalento.it (F.D.)

**Keywords:** β-oxidation, carnitine/acylcarnitine translocase, citrate carrier, fatty acid synthesis, hormonal regulation, nutritional regulation

## Abstract

The transport of solutes across the inner mitochondrial membrane is catalyzed by a family of nuclear-encoded membrane-embedded proteins called mitochondrial carriers (MCs). The citrate carrier (CiC) and the carnitine/acylcarnitine transporter (CACT) are two members of the MCs family involved in fatty acid metabolism. By conveying acetyl-coenzyme A, in the form of citrate, from the mitochondria to the cytosol, CiC contributes to fatty acid and cholesterol synthesis; CACT allows fatty acid oxidation, transporting cytosolic fatty acids, in the form of acylcarnitines, into the mitochondrial matrix. Fatty acid synthesis and oxidation are inversely regulated so that when fatty acid synthesis is activated, the catabolism of fatty acids is turned-off. Malonyl-CoA, produced by acetyl-coenzyme A carboxylase, a key enzyme of cytosolic fatty acid synthesis, represents a regulator of both metabolic pathways. CiC and CACT activity and expression are regulated by different nutritional and hormonal conditions. Defects in the corresponding genes have been directly linked to various human diseases. This review will assess the current understanding of CiC and CACT regulation; underlining their roles in physio-pathological conditions. Emphasis will be placed on the molecular basis of the regulation of CiC and CACT associated with fatty acid metabolism.

## 1. Introduction

Mitochondria are well-defined cytoplasmic organelles, which undertake multiple critical functions in the cell. In addition to oxidative phosphorylation (OXPHOS), a pathway in which nutrients are oxidized to form adenosine triphosphate (ATP), mitochondria are involved in several pathways including citric acid cycle, gluconeogenesis, fatty acid oxidation and lipogenesis, amino acid degradation and heme biosynthesis. They produce most of the cellular reactive oxygen species (ROS), buffer cellular Ca^2+^ and they initiate cellular apoptosis [1,2,3]. Moreover, mitochondria participate in cell communication and inflammation, and play an important role in aging, drug toxicity, and pathogenesis [4].

Mitochondria and cytosol are engaged in numerous metabolic processes which, due to enzyme compartmentalization, involve the exchange of metabolites among them.

Energy transduction in mitochondria requires the transport of specific metabolites across the inner membrane, achieved through mitochondrial carriers (MCs), a family of nuclear-encoded proteins sharing several structural features. Their common function is to provide a link between mitochondria and cytosol by facilitating the flux of a high number of metabolites through the permeability barrier of the inner mitochondrial membrane (IMM).

In humans, MCs are encoded by the *SLC25* genes, and some of them have isoforms encoded by different genes [4]. Until now, 53 mitochondrial carriers have been identified on the human genome and more than half have been functionally characterized [4].

Members of SLC25 family, mainly located in mitochondria, have been found in all eukaryotes; few of them are present in peroxisomes and chloroplasts [5]. *SLC25* genes are highly variable in size and organization, whereas their products are very similar sharing a tripartite structure composed by 100 amino acid repeats [4].

The complete sequence analysis of some MCs during the late 1990s showed that each domain contains two transmembrane α-helices, separated by hydrophilic regions, and a common signature motif which can be divided into a first part, P-X-D/E-X-X-K/R, and a second part as [D/E]GXXXX[W/Y/F][R/K]G [4]. The common structure is reflected in a similar function. To transport solutes across IMM, many MCs catalyze an exchange reaction: they have only one binding site, which is alternately exposed to the two opposite sides of the membrane. The substrate-induced conformational changes occur during the transition from cytosol to matrix and vice versa [4]. However, MCs do not adopt only antiport as transport; the carnitine-acylcarnitine translocase (CACT) catalyzes both unidirectional transport of carnitine and the carnitine/acylcarnitine exchange [6], whereas the uncoupling protein catalyzes uniport as the exclusive transport mode [7].

Functional characterization of MCs was carried out through their purification and reconstitution in artificial membranes, such as proteoliposomes. Studies on the transport activity performed with the liposomal systems show a dependence of the kinetic parameters on the lipid composition of the mitochondrial membrane, particularly on the cardiolipin (CL) levels [8]. CL interacts with a number of proteins and enzymes involved in fundamental mitochondrial bioenergetic processes [9]. Thus, CL is crucial for mitochondrial OXPHOS and for correct structure and function of IMM. It has been proposed that CL creates an environment protecting and stabilizing MCs in a functionally intact state [8].

**Figure 1:**
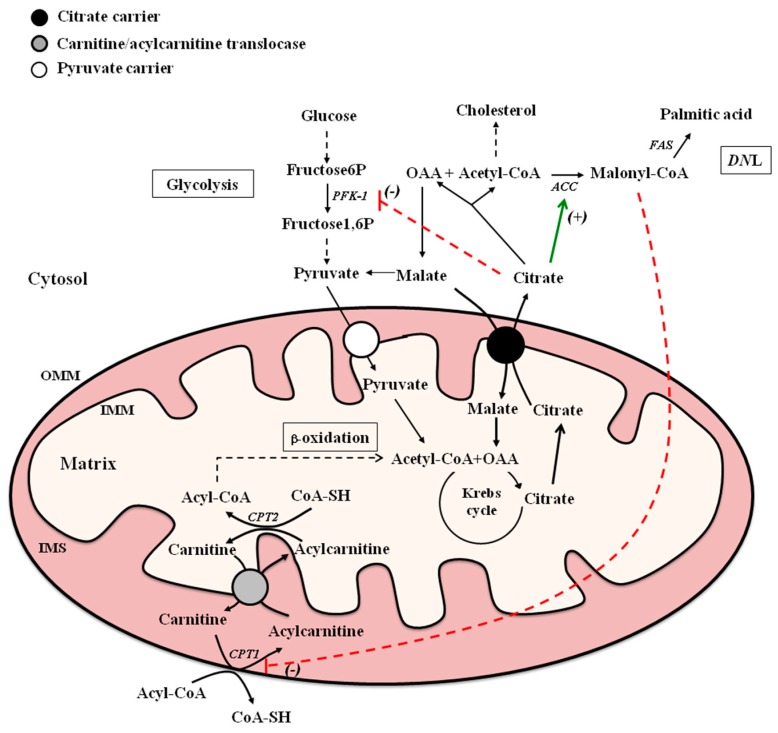
Schematic model of citrate carrier (CiC) and carnitine-acylcarnitine translocase (CACT) in lipogenesis and β-oxidation, and their metabolic interrelationship. Abbreviations: ACC, acetyl-CoA carboxylase; CoA-SH, coenzyme-A; CPT1, carnitine palmitoyltransferase 1; CPT2, carnitine palmitoyltransferase 2; *DN*L, *de novo* lipogenesis; FAS, fatty acid synthase; IMM, inner mitochondrial membrane; IMS, intermembrane space; OAA, oxaloacetate; OMM, outer mitochondrial membrane; PFK-1, phosphofructokinase-1. The green arrow and dashed red lines represent, respectively, positive (+) and negative (-) allosteric modulation of the indicated target enzymes.

MCs play a crucial role in intermediary metabolism. In this respect, citrate carrier (CiC) and CACT are two MCs mainly involved in fatty acid metabolism. CiC, encoded by *SLC25A1*, promotes the efflux of citrate from the mitochondria to the cytosol where citrate is cleaved by ATP-citrate lyase to oxaloacetate (OAA) and acetyl-coenzyme A (acetyl-CoA), which is used for fatty acid and sterol syntheses. CACT catalyzes the transport of fatty acids, in the form of acylcarnitine, into mitochondria, where they are oxidized by the enzymes of β-oxidation pathway (Figure 1). These MCs are mutually regulated to allow the synthesis and oxidation of fatty acids to take place at different times. The aim of this review is to summarize biochemical, molecular, and physio-pathological aspects of CiC and CACT.

## 2. Citrate Carrier (CiC) and Carnitine-Acylcarnitine Translocase (CACT): Mitochondrial Carriers in Fatty Acid Metabolism

Fatty acids perform several functions in cells; as components of triacylglycerols, they represent the main form of stored energy, and as constituents of phospholipids they play important structural roles, while some of them are also involved in intracellular signaling.

Fatty acid metabolism requires the involvement of both cytosolic and mitochondrial reactions (Figure 1).

The *de novo* lipogenesis (*DN*L) (*i.e.*, *de novo* fatty acid synthesis) takes place in the cytosol. The initiation of *DN*L occurs in the presence of high levels of blood glucose, indicating a sufficient energy intake. In this condition, the pancreas secretes insulin, which not only promotes the uptake of glucose from blood into the cells but also stimulates the synthesis of two enzymes of the *DN*L, acetyl-CoA carboxylase (ACC) and fatty acid synthase (FAS) [10]. These enzymes work in sequence to convert first acetyl-CoA to malonyl-CoA, in a reaction catalyzed by ACC, and then, by a series of reactions catalyzed by FAS, to produce palmitate, a saturated fatty acid with 16 carbon atoms. The condensation of malonyl, bound to acyl carrier protein (ACP), with acetyl-CoA by the ketoacyl-ACP synthase, is the first reaction catalyzed by FAS.

Acetyl-CoA utilized for *DN*L is mainly derived from carbohydrate metabolism. In this respect, after glucose conversion into pyruvate, the latter enters through its specific transporter into mitochondria, where it is converted in acetyl-CoA in a reaction catalyzed by pyruvate dehydrogenase. In the Krebs cycle, acetyl-CoA is then converted into citrate (tricarboxylate) after condensation with OAA. In a good energetic state, citrate is transported into the cytosol via CiC in exchange for malate (dicarboxylate) (the citrate/malate antiporter). The exchange is electroneutral being citrate efflux compensated by a contemporary efflux of a proton [4]. CiC transport activity is particularly high in the liver where active fatty acid synthesis occurs, and it is virtually absent in other tissues. CiC mRNA and/or protein levels are high in the liver, pancreas, and kidney, but are low or absent in the brain, heart, skeletal muscle, placenta, and lungs [11].

By the action of ATP-citrate lyase, cytosolic citrate is converted into OAA and acetyl-CoA, and this latter is used for the synthesis of fatty acids and cholesterol. OAA produced in the cytosol by ATP-citrate lyase is reduced to malate, which is converted to pyruvate via the malic enzyme with production of cytosolic NADPH plus H^+^ necessary for fatty acid and sterol syntheses. Moreover, citrate in the cytoplasm blunts glycolysis by inhibiting phosphofructokinase-1 (PFK-1), and positively modulates ACC, a key enzyme of the *DN*L pathway [12]. Additionally, the entry of malate into mitochondria in exchange for citrate stimulates OXPHOS [13].

Differently from fatty acid synthesis, fatty acid oxidation occurs in mitochondria. The signal for fatty acid oxidation begins with the secretion of glucagon or, in some cases, epinephrine [14]. These hormones stimulate enzymes that clip off fatty acids from triacylglycerol molecules. During fatty acid oxidation, two-carbon units are sequentially cleaved from the fatty acid chain, as acetyl-CoA, which then enters the Krebs cycle.

β-Oxidation can occur when fatty acids cross the IMM. This is achieved through CACT encoded by *SLC25A20* [15]. The *CACT* gene is differently expressed in human tissues. High levels of transcripts are found in liver, heart and skeletal muscle, where β-oxidation is essential for energy production; much lower levels are observed in other tissues, such as brain, placenta, kidney, pancreas and lung [16].

This carrier belongs to the carnitine palmitoyl transferase (CPT) system, the major site of control of fatty acid β-oxidation, which transports cytosolic long chain fatty acids (LCFA) in the form of esters of CoA-SH (LCFA-CoA) into the mitochondrial matrix for their oxidation [17]. Three different proteins are involved in the carnitine-dependent transport: carnitine palmitoyl transferase 1 (CPT1), CACT and carnitine palmitoyl transferase 2 (CPT2). The acyl-CoAs, products of the cytosolic activation of fatty acids, are transformed into the corresponding carnitine esters by CPT1 localized in the outer mitochondrial membrane (OMM) [18]. Then, acyl-carnitine permeates the IMM by CACT and reacts with a matrix pool of CoA-SH in a reaction catalyzed by CPT2 on the inner face of the IMM. The reformed acyl-CoA then enters the β-oxidation pathway, while the released carnitine returns to the extramitochondrial compartment.

This pathway is the major source of energy for heart and skeletal muscles during fasting and physical exercise. Besides the exchange, CACT performs unidirectional transport of carnitine across IMM but to a much lower rate (about one tenth of the exchange); uniport of carnitine balances the matrix carnitine pool, a prerequisite for optimal carnitine/acylcarnitine activity [6].

Malonyl-CoA, the first committed intermediate in the pathway of fatty acid synthesis, represents an allosteric modulator of fatty acid oxidation. In the fed state, when insulin/glucagon ratio is high, hepatic lipogenesis is active, the concentration of malonyl-CoA rises and becomes sufficient to inhibit CPT1 [19], the enzyme catalyzing the rate-limiting step in fatty acid oxidation. In this condition, while fatty acid oxidation is low or absent, lipogenesis is up-regulated, being malonyl-CoA a substrate for FAS.

Conversely, in ketotic states (low insulin/glucagon ratio) carbon flow through glycolysis and ACC diminishes, the malonyl-CoA level falls. In this setting, CPT1 is disinhibited, and incoming fatty acids readily undergo β-oxidation with accelerated production of ketone bodies.

It follows that fatty acid oxidation and fatty acid synthesis fluctuate reciprocally with changes in malonyl-CoA levels [20].

## 3. CiC and CACT Involvement in Pathological States

The human *SLC25A1* gene, encoding for CiC, maps on the chromosome 22.q11.2. Micro deletions involving 22q11.2 have been associated with developmental disorders known as DiGeorge syndrome, velo-cardio-facial syndrome, and a subtype of schizophrenia [21]. However, direct evidence of the relevance of *SLC25A1* haploinsufficiency in these syndromes so far has not been found.

Recessive mutations in *SLC25A1* with impaired mitochondrial citrate efflux are found in patients with combined d-2- and l-2-hydroxyglutaric aciduria (d,l-2-HGA), a disease characterized by epileptic encephalopathy, respiratory insufficiency, developmental arrest and early death [22]. Recently, it has been reported that null/missense *SLC25A1* mutations, besides classic clinical features of d,l-2-HGA, showed marked facial dysmorphism and prominent lactic acidosis [23]. Moreover, *SLC25A1* knockdown has been related to pre-synaptic nerve terminal abnormalities and neuromuscular junction impairment [24].

Increased levels of CiC have been found in human cancers, while inhibition of CiC activity showed anti-tumor activity, and *SLC25A1* gene has been implied in epigenetic regulation or cancer biology [22]. Citrate exported from mitochondria via CiC and its downstream metabolic intermediate, acetyl-CoA, are necessary for cytokine induced inflammatory signals [25] and CiC acetylation plays a key role in the production of inflammatory mediators in activated immune cells [26].

Low levels of CiC activity and expression were measured in primary biliary cirrhosis, together with a low synthesis of fatty acids. The impaired CiC activity and expression was almost completely prevented by treatment with Silybin, an extract of silymarin with antioxidant and anti-inflammatory properties [27].

Several lines of evidence suggest the involvement of CiC in the development of liver steatosis, which is characterized by accumulation of lipid droplets in hepatocytes. This is partially due to the increased lipogenic gene expression, linked to the Unfolded Protein Response (UPR) pathway [28,29]. Indeed, in HepG2 and in BRL-3A cells, CiC expression was increased upon the induction of endoplasmic reticulum (ER) stress and the consequent activation of UPR pathway [29].

*SLC25A20* gene maps on the chromosome 3p21.31 [30], spread over 42 kb, consist of nine exon and eight intron and encode for CACT, a protein of 301 amino acids [31]. CACT deficiency, described for the first time in 1992 [32], may present two different phenotypes: the most common with an early onset in the neonatal period and a milder form with onset in infancy or, less frequently, in childhood.

A mutation in the *SLC25A20* gene was firstly individuated by Huizing [15]. Since then, 35 others mutations have been identified in the *SLC25A20* gene [33]. Usually, disease-triggering mutations affect residues of the carrier signature motif. Deficiency of CACT activity can also be a consequence of an elongation in the C-terminal portion of the protein [15]. In any case, defective CACT results in decreased carnitine/acylcarnitine transport and impaired fatty acid β-oxidation. The clinical consequences of such alterations may involve hypoglycaemia, hyperammonaemia, cardiomyopathy, liver failure and encephalopathy [33].

## 4. Hormonal Regulation of CiC and CACT Activity and Expression

### 4.1. Thyroid Hormones

Thyroid hormones influence synthesis, mobilization and degradation of lipids. In this regards, thyroid hormones have been demonstrated to affect the activities of MCs directly involved in lipid metabolism [34,35,36,37,38].

Modulation of CACT activity by thyroid hormones has been reported by Paradies *et al.* [35]. In this study, an increased rate of palmitoylcarnitine/carnitine exchange in heart mitochondria from hyperthyroid rats has been demonstrated. Conversely, the hypothyroid state reduced fatty acid oxidation in rat heart mitochondria due to a decreased CACT activity which was restored to normal levels after 3,3′,5-triiodo-l-thyronine (T3) administration [36]. Analysis of kinetic parameters of CACT demonstrated that both hyper- and hypothyroidism significantly affected *V*max without changing *K*m value and the changes in heart CACT activity were ascribed to changes in CL level [36] (Table 1).

Moreover, T3 stimulated in the liver the transcription of *CPT1* gene in coordination with other genes involved in fatty acid oxidation [39,40], binding to a thyroid hormone response element (TRE), present in CPT1 promoter [41].

Liver CiC activity was demonstrated to be significantly stimulated in hyperthyroid with respect to euthyroid rats [34]. Changes in the mitochondrial membrane lipid composition and in the amount of CL strictly associated to CiC were reported to be involved in the T3-induced increase of CiC activity [34] (Table 1).

Conversely, hepatic CiC activity and expression were decreased, together with the expression of lipogenic genes *ACC* and *FAS*, in hypothyroid rats [37]. A decrease of mRNA abundance and protein level, due to a lower transcription rate and splicing of CiC pre-mRNA, were responsible for the impaired CiC activity in the hypothyroid state [38].

It has been demonstrated that T3 is able to directly increase ACC and FAS mRNA transcription through interaction with TRE located on the respective gene promoters [42,43]. To date, a TRE has not been found in the CiC promoter. Thus, the mechanism of T3 effect on *CiC* gene expression has not been yet fully clarified. It is plausible that T3 may regulate *CiC* gene expression through Sterol Regulatory Element-Binding Protein-1 (SREBP-1), which is considered the master transcription factor involved in the regulation of lipogenic gene expression [44]. Indeed, T3 affecting SREBP-1 expression [45] could regulate *CiC* gene expression through the SREBP-1 binding site found on human [46] and rat [47] CiC promoters.

Thus, the T3 activation of liver fatty acid synthesis and oxidation can support the establishment of a futile cycle.

### 4.2. Diabetes and Insulin

#### 4.2.1. Type 1 Diabetes

A decrease in CiC activity was measured in experimental type 1 diabetic rats [48,49] and kinetic studies showed a reduction in *V*max and almost unchanged *K*m of CiC protein [48] (Table 1). The observed reduction of CiC activity was mainly ascribed to a reduced level of both CiC mRNA and translated protein. The reduction of CiC expression in diabetic rats is attributed either to transcriptional and post-transcriptional gene regulation. Indeed, both the transcriptional rate of *CiC* gene and the splicing reaction of CiC pre-mRNA decreased in nuclei from diabetic rats [50]. Injection of insulin to diabetic rats increased hepatic CiC activity and protein level to values higher than those measured in control animals [48].

Several lines of evidence suggest that insulin regulation of CiC is mediated by SREBP-1 [50]. Sites for SREBP-1a transactivation of *CiC* gene have been characterized on human and rat *CiC* promoter, at −1696 bp [46] and −67 bp [47], respectively. In rat hepatocytes cultured in the absence of insulin, a reduction of *CiC* promoter activity was observed as a consequence of a decrease of SREBP-1 expression [50]. Furthermore, the binding of SREBP-1 to the *CiC* promoter was reduced in diabetic rats with respect to control ones and it was restored to the control values after insulin treatment [50]. Overall, these results emphasize that insulin participates in the regulation of the functional levels of CiC in rat liver.

The activity and the expression of CiC were increased in liver of rats fed on a high carbohydrate diet [51]. In liver from diabetic rats, correction of hyperglycemia with phlorizin restored CiC activity and protein level to values measured in control animals [48] (Table 1). However, the molecular mechanism/s by which hyperglycemia affects CiC expression is not so far understood.

#### 4.2.2. Type 2 Diabetes

Increased plasma levels of acylcarnitines have been associated with type 2 diabetes and insulin resistance [52,53]. Acute carnitine administration is able to improve peripheral insulin sensitivity in non-insulin-dependent diabetic patients [54] and to relieve glucose intolerance in obese rodents [55]. CACT expression was up-regulated in pancreatic islets of diabetic obese mice [56]. Moreover, CACT levels decreased in the kidney of diabetic rats [57] and in the muscle of insulin-resistant patients [58]. These data highlight the relevance of fatty acid accumulation in the muscle for the etiology of insulin resistance. No effect of type 2 diabetes on rat liver CiC activity has been observed [59].

#### 4.2.3. Insulin Secretion

Studies have reported that both CiC and CACT may be involved in insulin responses.

CACT down-regulation by RNA interference enhances the insulin secretion in murine pancreatic Langerhans islets [60]. On the contrary, reduction in CiC expression by CiC-specific siRNA inhibited glucose-stimulated insulin secretion in normal rat pancreatic islets [61].

Recently, a study provided evidence that the inhibition of CiC by the specific substrate analog 1,2,3-benzenetricarboxylate resulted in a reduction of glucose-stimulated insulin secretion and autocrine insulin secretion by sperm [62]. These data furnish a new site of action for CiC in the regulation of sperm energetic metabolism to sustain capacitation process and acrosome reaction.

## 5. Nutritional Regulation of CiC and CACT Activity and Expression

### 5.1. Starvation

During fasting, an up-regulation of genes involved in fatty acid catabolism is observed [63,64]. This metabolic response is mediated by Peroxisome Proliferator-Activated Receptor α (PPARα), a transcriptional factor activated by free fatty acids. Different hypotheses have been proposed on the origins of the free fatty acids activating PPARα in the fasting state [65,66]. In response to fasting, PPARα stimulates the transcription of a large number of genes encoding for proteins involved in fatty acid catabolism, such as fatty acid transporters, fatty acid binding proteins, acyl-CoA synthase, CPT1 and CPT2 [63,64]. PPARα is also a strong activator of *CACT* gene expression [67]. CACT [68] as well as CACL, a CACT-like protein expressed in the brain [69], are up-regulated during fasting (Table 2). Anti-hyperlipidemic drugs, such as statins and fibrates, up-regulate expression of CACT [67,68]. This would be expected to enhance fatty acid oxidation and, therefore, may contribute to the lipid-lowering effects of these agents. Fibrates exert their effect by binding to PPARα [70], statins by inhibiting the Rho-signaling pathway [71], and retinoic acid by activating PPARα-RXRα heterodimer when bound to the PPAR responsive element (PPRE) [72].

On the other hand, a noticeable reduction of CiC activity has been observed in starved rats [73,74] (Table 2). No change in the membrane lipid composition and fluidity was detected in mitochondria from liver of starved rats suggesting that the reduction of CiC activity could be ascribed to modifications of CiC expression [73]. When compared to fed animals, in starved rats a considerable reduction of CiC mRNA abundance was observed. The reported reduction was caused by an increment in mRNA turnover, suggesting that starvation accelerates the degradation of CiC mRNA [74]. Altogether, the up-regulation of CACT and the down-regulation of CiC are aimed at increasing fatty acid oxidation, the major metabolic pathway for energy supply in the fasting state.

### 5.2. Saturated and Unsaturated Fatty Acids

It is well known that fatty acids, and in particular polyunsaturated fatty acids (PUFA), are potent regulators of cell metabolism. Fatty acids can influence hormonal signaling events by modifying membrane lipid composition, but they also have a direct effect on the regulation of genes mainly involved in carbohydrate metabolism and lipogenesis [75] as well as in fatty acid β-oxidation [76].

CACT activity and expression were affected by diets supplemented with different fat types [77,78] (Table 2). Fish oil (FO)-enriched diets, rich in ω-3 PUFA, increased rat liver CACT mRNA level and activity, whereas olive oil (OO)-enriched diets, rich in monounsaturated fatty acids (MUFA), reduced CACT mRNA level when compared to beef tallow (BT) rich in saturated fatty acids (SFA). ω-6 PUFA-supplemented diets did not affect CACT activity and expression [78] analogously to what was reported for CPT1 and CPT2 [77,78]. Recently, an up-regulation of CACT mRNA in FO-fed grass carp has also been reported [79].

FO treatment increased the transcriptional rate of CACT mRNA. On the other hand, OO modulated the splicing of the last intron of CACT pre-mRNA, and the rate of 3′-end formation [78]. It has been demonstrated that ω-3 PUFA are important positive regulators of PPARα [80], a transcriptional factor which positively regulates *CACT* gene expression. Notably, a functional PPRE in the CACT promoter has been identified [67,68].

Differently from CACT, several studies (Table 2) reported that CiC activity and expression were inhibited in liver of rats fed on a diet supplemented (15%) with FO [81,82] or safflower oil [83,84], rich in ω-3 or ω-6 PUFA, respectively. When compared with control rats, C_18:2_ conjugated linoleic acids did not affect CiC activity [85], whereas CiC activity was lower in rats fed on diets enriched in oleic (C_18:1_ cis) or elaidic (C_18:1_ trans) acid [86]. A significant decrease in citrate transport was also observed when rats were fed on a diet containing a small percentage (2.5%) of FO for a relatively short period of treatment (2–3 weeks) [87]. The decrease was more pronounced when ω-3 PUFA were administered in the form of krill oil [87]. Corn oil and pine nut oil, rich in ω-6 PUFA, were able to reduce the activities of hepatic CiC and the cytosolic lipogenic enzymes in mice [88] (Table 2).

Feeding rats for three weeks on a diet supplemented with FO significantly decreased liver CiC activity when compared to SFA [81]. A study in which rats were fed on diets enriched in OO, FO or BT showed that CiC transcription rate, mRNA turnover and RNA processing were decreased only upon FO-feeding [82]. These data indicate that FO administration regulates *CiC* gene at transcriptional and post-transcriptional levels, whereas BT- and OO-feeding affects neither CiC activity nor *CiC* gene expression [82]. Inhibition of activity and expression of CiC, as well as of lipogenic enzymes ACC and FAS, were found in the liver of rats fed on a high fat diet (HFD) enriched with two different doses of SFA for one week [58]. However, this inhibition was progressively attenuated in long feeding experiments suggesting that the effects of HFD enriched in SFA on CiC activity and expression was time- and dose-dependent [58].

Diets enriched with PUFA of the ω-6 or ω-3 series strongly reduce hepatic lipogenesis both in human and in animal models [75]. Although changes in the lipid composition have been found in the membrane of hepatic mitochondria from PUFA-fed rats, the decreased CiC activity was mainly ascribed to a reduction of *CiC* gene expression, as shown by *in vivo* [81,82,83] and *in vitro* studies [47].

Several molecular events were reported for the PUFA-mediated reduction of CiC expression. Dietary PUFA administration reduces both the transcriptional rate and the splicing process of CiC pre-mRNA, whereas no change in the estimated half-life of the transcript was found [82,84]. Taken together, the aforementioned reports indicate that ω-3 and ω-6 PUFA-supplemented diets down-regulate hepatic *CiC* gene expression by both transcriptional and post-transcriptional mechanisms [82,84].

Two distinct transcription factors, SREBP-1 and PPARs, mediate the regulation of *CiC* gene expression by PUFA.

It has been demonstrated that PUFA of the ω-3 or ω-6 series reduce the transcriptional activity of SREBP-1. In HepG2 [46,47] and in H4IIE hepatoma cell lines [47] SREBP-1 mRNA and protein levels are reduced by the ω-3 PUFA docosahexaenoic acid (DHA, C_22:6_). Taking into account that CiC promoter is activated by SREBP-1 [47], the reduction of CiC mRNA level observed in DHA-treated cells was ascribed to the PUFA-mediated inhibition of *CiC* promoter transactivation by SREBP-1 [47].

Different results have been obtained in the non tumoral BRL-3A hepatic cell line treated with DHA [89]. A strong decrement of CiC expression and *CiC* promoter activity was observed in BRL-3A treated with 50 µM DHA. However, both CiC expression and CiC promoter activity increased in hepatocytes treated with concentrations of DHA higher than 50 µM. As PUFA are natural ligands of PPARα, these findings were ascribed to the transactivation of CiC promoter by this nuclear receptor [89]. This hypothesis was supported by the induction of *CiC* promoter activity in BRL-3A cells upon PPARα/RXRα overexpression or treatment with WY-14,643, a specific PPARα agonist [89]. Moreover, a functional PPRE has been identified at −625 bp of the *CiC* promoter [89]. Since PPARα is the master regulator of genes for β-oxidation enzymes, the physiological role of *CiC* gene transactivation by this transcriptional factor is not yet fully understood. However, an implication of CiC transcriptional activation by PPARα in gluconeogenesis has been suggested [89]. An increase in CiC mRNA abundance and protein level was observed during the induction of murine 3T3-L1 cell differentiation into mature adipocytes, as well as in cells treated with rosiglitazone, a PPARγ agonist, suggesting the involvement of CiC in adipogenesis [89,90].

## 6. Conclusions

In the last years, the molecular studies on the expression of carrier genes and the functional characterization of their promoters have provided information about specific functions of different MCs. *CACT* and *CiC*, members of *SLC25* family of MCs, are involved in fatty acid metabolism. They have been functionally characterized and their regulation at the transcriptional level has been investigated.

Although much progress has recently been made in the study of the regulation of *CiC* and *CACT* gene expression, underlying mechanisms in different species, tissues, metabolic and hormonal states are not completely understood.

While the effects of different nutritional and hormonal states on the activity and expression of the cytosolic lipogenic enzymes as well as of the mitochondrial enzymes involved in fatty acid oxidation have been deeply investigated, to date a similar work has not been done on CiC and CACT. Considering that the rate of efflux or influx of metabolites through IMM could regulate cellular pathways, the knowledge of the mechanisms by which different nutritional and hormonal factors can control CiC and CACT functions is crucial. These studies may also provide insight into the interconnection existing between catabolic and anabolic pathways inside the cells.

In the future, it could be interesting to investigate whether transcriptional regulation of *CiC* and *CACT* is relevant in diseases associated with insulin signal deregulation, such as obesity and metabolic syndrome. In this respect, in light of the role played by acylcarnitine accumulation in metabolic syndrome [60], the development of therapeutic strategies to regulate CACT activity might furnish valid approaches to the management of syndromes associated with altered fatty acid oxidation.

It is important to note that cytoplasm citrate, conveyed by CiC, is cleaved to acetyl-CoA which is not only the precursor for fatty acid and sterol biosynthesis but it is also the universal donor for protein and histone acetylation [12]. It is worth underlining that CiC expression is increased in cancer cells, in which high levels of acetyl-CoA are required for both lipid synthesis and histone acetylation [12].

Furthermore, the N-terminal (Nt) acetylation of most cellular proteins plays a crucial role in different cellular pathways including apoptosis, regulation of protein degradation through recruitment of ubiquitin ligases [91], prevention of protein translocation from the cytosol to the endoplasmic reticulum (ER), protein complex formation and membrane attachment of small GTPases involved in organelle trafficking [91]. Since it has been demonstrated that the level of acetyl-CoA can regulate the abundance of acetylated proteins, it might be interesting to study: (i) the potential role of CiC and CACT in these cellular processes and (ii) if CACT activity, similarly to CiC, can be regulated by acetylation reactions. These studies could open up interesting new fields on the mechanisms involved in the regulation of lipid metabolism in the cell.

## Figures and Tables

**Table 1:** Effect of the hormonal status on activity, kinetics, protein and mRNA levels of CACT and CiC.

Hormonal Status	Carrier	Activity	*K*m	*V*max	Protein	mRNA	References
Hyperthyroidism	CACT	+43%	↔	↑			[35]
Hypothyroidism	CACT	−41%	↔	↓			[36]
Hypothyroidism	CiC	−60%	↔	↓	−35%	−30%	[37]
Hyperthyroidism	CiC	+43%	↔	↑			[34]
Streptozotocin-induced diabetes (1–8 weeks)	CiC	−31% ÷ −51%					[49]
Streptozotocin-induced diabetes (3 weeks)	CiC	−35%	↔	↑	−37%	−35%	[48]

↔: no change; ↓: decrease; ↑: increase.

**Table 2:** Effect of the nutritional status on activity, kinetics, protein and mRNA levels of CACT and CiC.

Treatment	Carrier	Activity	*K*m	*V*max	Protein	mRNA	References
Fasting	CACT					+60%	[68]
	CACT				↑	↑	[69]
	CiC	−40%				−35%	[73,74]
ω-6 PUFA							
15% safflower oil for 3 weeks	CACT	↔	↔	↔	↔	↔	[78]
15% safflower oil for 3 weeks	CiC	−40%	↔	↓	−30%	−35%	[83]
15% safflower oil for 1–4 weeks	CiC	−50%				−35%	[84]
7.5% corn oil for 8 weeks	CiC	−60%			−70%		[88]
7.5% pine nut oil for 8 weeks	CiC	−40%					
ω-3 PUFA							
15% fish oil for 3 weeks	CACT	+50%	↔	↑	+60%	+70%	[78]
15% fish oil for 3 weeks	CiC	−60%	↔	↓	−50%	−40%	[81,82]
2.5% fish oil for 3 weeks	CiC	−30%	↔	↓	−30%	↔	[87]
2.5% fish oil for 6 weeks	CiC	−65%	↔	↓	−70%	−30%	[87]
2.5% krill oil for 6 weeks	CiC	−65%	↔	↔	−70%	−30%	[87]
CLA							
2.25% CLA for 2 weeks	CiC	↔					[85]
MUFA							
15% olive oil for 3 weeks	CACT	−10%	↔	↓	−20%	−20%	[78]
15% olive oil for 3 weeks	CiC	↔			↔	↔	[82]
14% oleic acid for 2 weeks	CiC	−22%					[85]
9.5% elaidic acid for 2 weeks	CiC	−36%					[85]
SFA							
20.2% SFA for 1 week	CiC	−54%	↔	↓	−40%	−30%	[58]
35.2% SFA for 1 week	CiC	−80%	↔	↓	−60%	−70%	[58]
Carbohydrate							
70% carbohydrate for 1 week	CiC	+20%	↔	↑	+20%	+45%	[58]

↔: no change; ↓: decrease; ↑: increase. CLA = conjugated linoleic acids; MUFA = monounsaturated fatty acids; PUFA = polyunsaturated fatty acids SFA = saturated fatty acids.
